# Unexpected complete remission after recurrence in pancreatic cancer: a case report on comprehensive multimodal therapy

**DOI:** 10.3389/fonc.2025.1582673

**Published:** 2025-10-17

**Authors:** Shangyou Zheng, Huimou Chen, Chonghui Hu, Tianhao Huang, Qing Lin, Honghui Jiang, Rufu Chen, Rihua He

**Affiliations:** ^1^ Department of Pancreas Center, Department of General Surgery, Guangdong Provincial People’s Hospital (Guangdong Academy of Medical Sciences), Southern Medical University, Guangzhou, Guangdong, China; ^2^ Department of Oncology, Sun Yat-sen Memorial Hospital of Sun Yat-sen University, Guangzhou, China

**Keywords:** PDAC, resectable pancreatic cancer, chemotherapy, tumor recurrence, pseudoprogression

## Abstract

Pancreatic ductal adenocarcinoma (PDAC) is among the most aggressive malignancies of the digestive tract, with a 5-year survival rate below 13%. It often infiltrates surrounding tissues early, making it challenging to distinguish primary pancreatic involvement from adjacent malignancies on imaging. Despite advancements in surgery, chemotherapy, and radiotherapy, PDAC still carries a high risk of recurrence. Here, we present a case initially diagnosed by imaging and biopsy as a duodenal malignancy. The patient underwent robotic-assisted laparoscopic pancreaticoduodenectomy, and final pathology confirmed moderately differentiated PDAC. Postoperative treatment with eight cycles of a modified AG (GN) regimen achieved 17 months of tumor-free survival. Following the detection of peritoneal metastases, the patient received six months of NALIRIFOX therapy and achieved complete remission by the ninth month after recurrence. This case underscores the critical role of surgery and adjuvant chemotherapy in resectable PDAC. It also highlights the importance of vigilant postoperative surveillance for early detection of recurrence and emphasizes the concept of pseudoprogression when interpreting imaging findings.

## Introduction

Pancreatic ductal adenocarcinoma (PDAC) ranks among the most aggressive malignancies, with a 5-year survival rate below 13% and a rising incidence (1). Although surgical resection and adjuvant chemotherapy are standard treatments, PDAC frequently recurs, reflecting its poor prognosis (2, 3). Despite advances in surgical techniques and chemotherapeutic regimens, long-term survival in PDAC remains low, largely attributable to the early metastatic nature of the disease and its resistance to conventional therapy.

Although imaging tests (e.g., CT, MRI, ultrasound) are widely used for diagnosing pancreatic cancer, they have notable limitations, particularly in detecting small tumors or those located in the pancreatic tail (4, 5). Furthermore, when the cancer invades adjacent tissues, imaging may not definitively establish whether the primary tumor originates in the pancreas or elsewhere. Pancreatic cancer recurrence and metastasis are both common and insidious, often progressing rapidly. Evaluating tumor progression in this malignancy is challenging because conventional imaging techniques—such as computed tomography (CT) and magnetic resonance imaging (MRI)—frequently fail to detect early recurrences or micrometastases due to their limited resolution and the desmoplastic tumor microenvironment (6). Moreover, although serum biomarkers like CA 19–9 are valuable for monitoring treatment response, they can be unreliable in patients with Lewis antigen-negative phenotypes (6). Post-treatment follow-up is critical, yet its implementation is often inconsistent. While current guidelines recommend regular imaging and CA 19–9 surveillance, the optimal frequency and duration vary widely across institutions (7). In addition, the psychological and logistical burdens of frequent follow-ups can adversely affect patient adherence. A more structured, patient-centered follow-up strategy—integrating advanced imaging techniques and molecular profiling—could improve early detection of recurrence and enable timely intervention (7).

Peritoneal metastases represent a frequent recurrence pattern in PDAC and pose a significant therapeutic challenge due to the inherent difficulties in delivering drugs within the peritoneal cavity (8, 9). Although these metastases generally indicate a poorer prognosis, gemcitabine-based chemotherapy regimens remain the cornerstone for treating advanced and recurrent PDAC (10). Recent advancements in chemotherapy have further improved patient outcomes. Notably, the NALIRIFOX regimen—which combines liposomal irinotecan (nal-IRI), 5-fluorouracil (5-FU), leucovorin, and oxaliplatin—has emerged as a promising therapeutic option. In fact, the 2023.V2 edition of the NCCN Clinical Practice Guidelines formally recommends NALIRIFOX as a first-line treatment for pancreatic cancer (11). Moreover, emerging evidence suggests that second-line therapies and more intensive regimens can offer substantial clinical benefits even in recurrent settings (11). Therefore, timely detection and appropriate management strategies are essential for improving patient outcomes.

In this report, we present a patient with PDAC who initially underwent a pancreaticoduodenectomy followed by adjuvant chemotherapy. Seventeen months later, the patient developed peritoneal metastases; however, subsequent treatment led to a clinical complete remission (CR). This case exemplifies a comprehensive management strategy—from diagnosis and surgery to adjuvant and salvage therapy—and underscores the potential for successfully treating recurrent PDAC with peritoneal metastases. Importantly, it highlights the necessity of continued, individualized treatment even after disease recurrence.

## Case presentation

**Figure 1:**
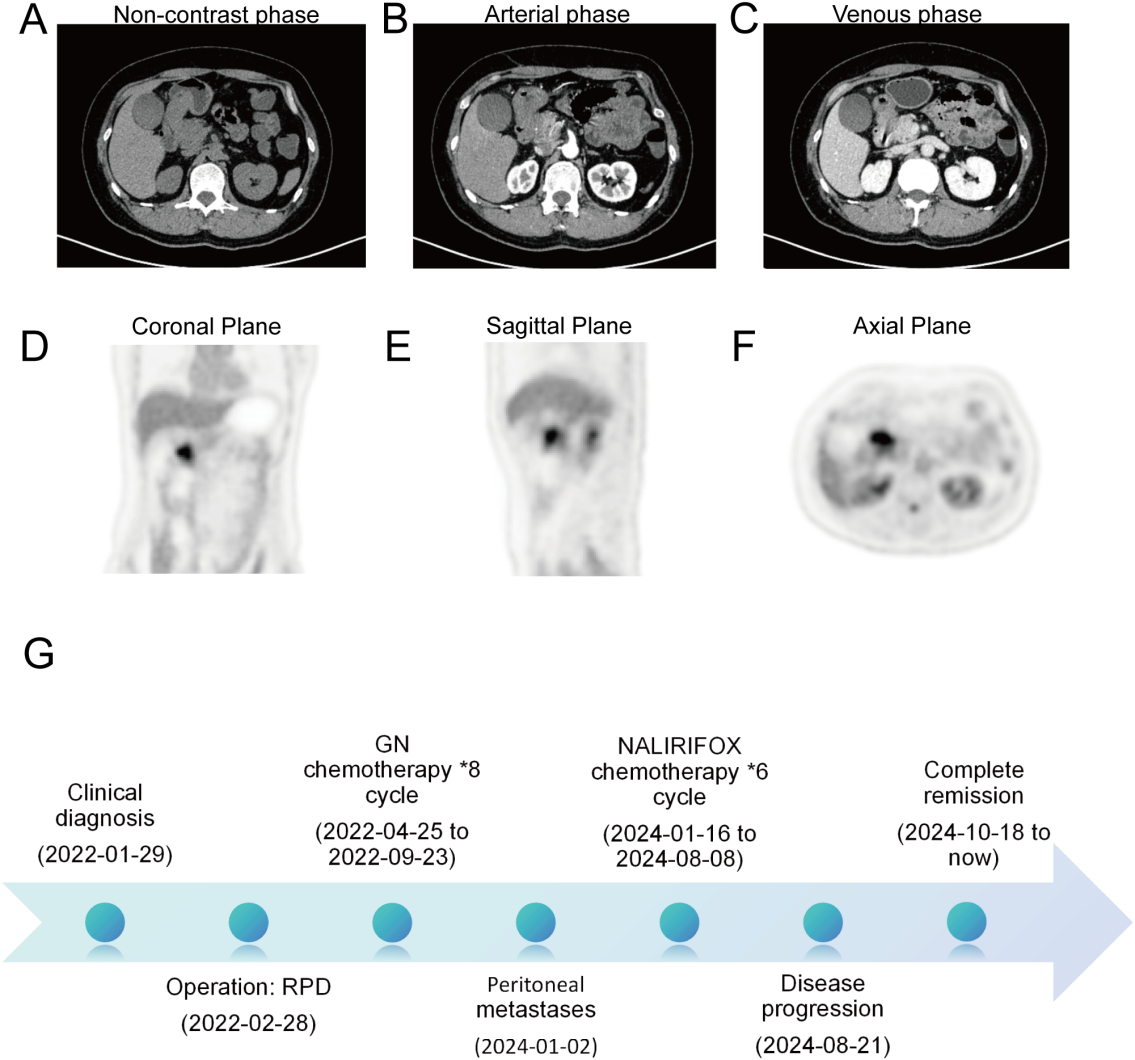
Preoperative PET/CT images of the patient. **(A)** Non-contrast phase of CT. **(B)** Arterial phase of CT. **(C)** Venous phase of CT. **(D)** Coronal plane of PET/CT. **(E)** Sagittal plane of PET/CT. **(F)** Axial plane of PET/CT. **(G)** Comprehensive summary flow chart of treatment.

**Figure 2:**
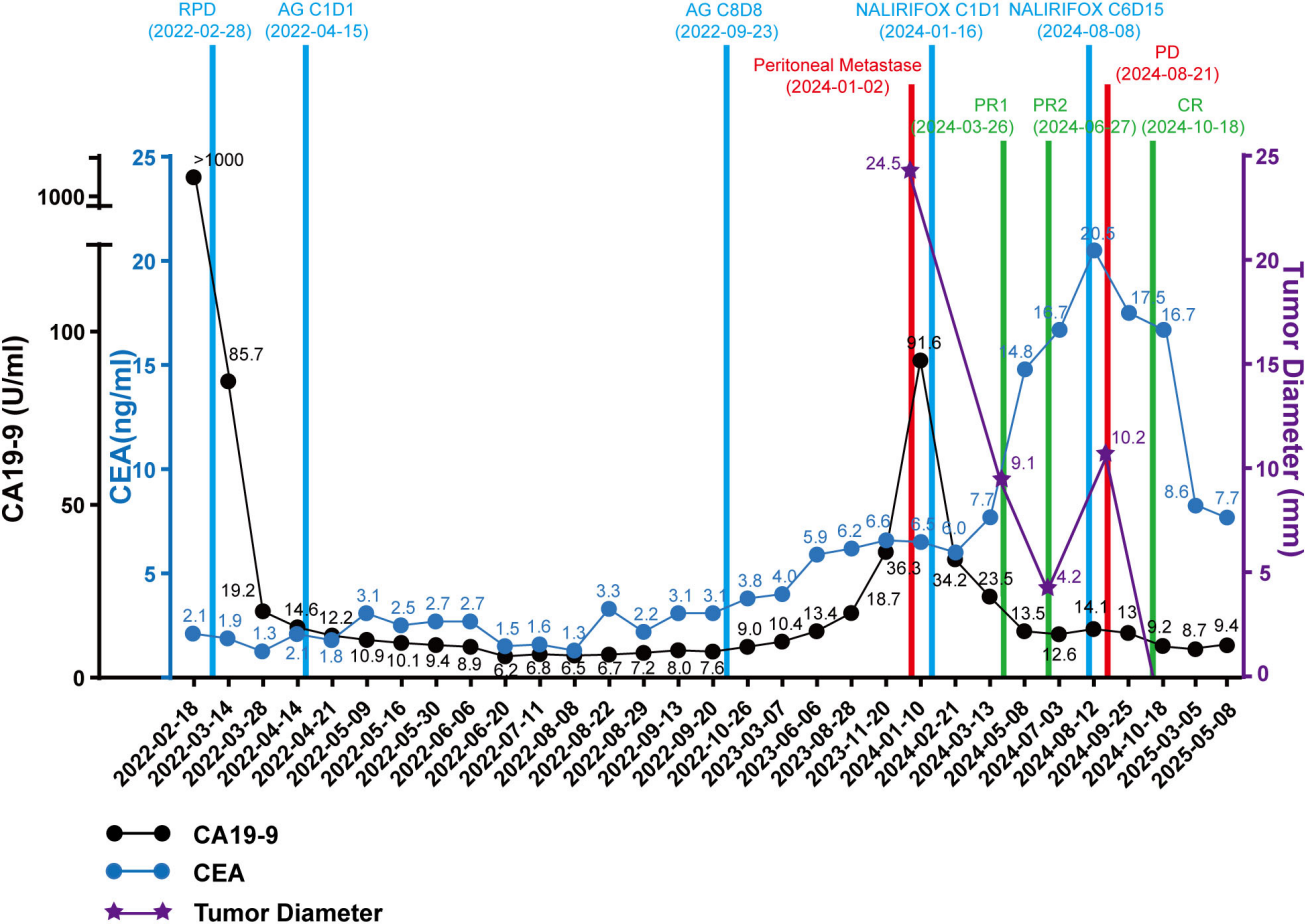
Treatment history and tumor indicators of the patient. Treatment history, CA19-9, CEA and tumor diameter of the patient.

A 49-year-old female presented with a two‐month history of upper abdominal bloating accompanied by a 3‐kg weight loss. She had no significant past medical or family history. Endoscopic ultrasound (EUS) revealed a mucosal elevation in the descending duodenum (**Supplementary Figure S1**), and histopathologic examination of the biopsy specimen confirmed a moderately differentiated adenocarcinoma. Positron emission tomography/computed tomography (PET/CT) demonstrated marked eccentric, irregular thickening of the duodenal bulb wall, with a maximal thickness of approximately 1.4 cm spanning a length of about 3.6 cm. The lesion showed mild to moderate enhancement with a coarse external serosal layer. Surrounding fat planes were indistinct, and multiple small nodular shadows were noted. Increased FDG uptake was observed within the lesion (SUVmax 8.2). Additionally, the margin of the adjacent pancreatic head was poorly defined with slightly reduced enhancement, while no enlarged lymph nodes or abnormal FDG uptake were identified in the mesenteric and retroperitoneal regions (**Figures 1A–F**). Laboratory investigations revealed an elevated serum carbohydrate antigen 19-9 (CA 19-9) level exceeding 1000 U/ml (**Figure 2**), while alpha-fetoprotein (AFP), carcinoembryonic antigen (CEA), and carbohydrate antigen 125 (CA 125) remained within normal limits. Based on imaging and tumor marker findings, a preliminary diagnosis of duodenal cancer was made—with suspicion of adjacent fat space invasion and potential involvement of the pancreatic head.

**Figure 3:**
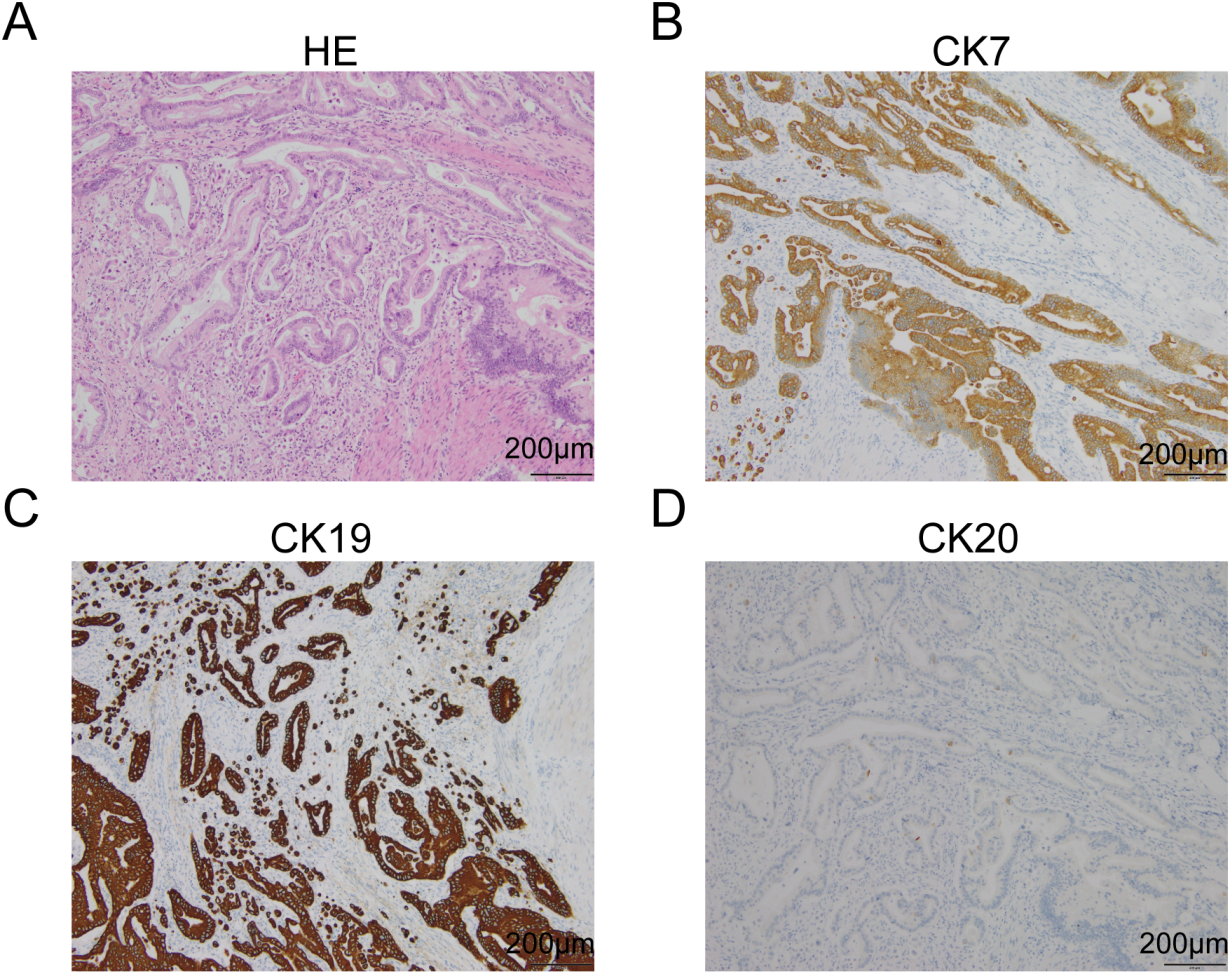
Postoperative pathology of the patient. **(A)** HE staining of postoperative pathological tissues. **(B)** CK7 IHC of postoperative pathological tissues. **(C)** CK19 IHC of postoperative pathological tissues. **(D)** CK20 IHC of postoperative pathological tissues.

On February 28, 2022, the patient underwent a robot-assisted laparoscopic pancreatoduodenectomy (RPD) with skeletonisation of groups 8, 12, 13, 14, 16 and 17 lymph nodes (These groups encompass nodes along the common hepatic artery, hepatoduodenal ligament/portal vein (group 12), posterior pancreatic head (group 13), superior mesenteric artery (group 14), para aortic region (group 16) and anterior pancreatic head (group 17).). Postoperative pathology confirmed a moderately differentiated pancreatic ductal adenocarcinoma (**Figure 3A**) with a maximum tumor diameter of approximately 2.8 cm. The tumor demonstrated nerve bundle invasion and penetrated the full thickness of the duodenal wall to the mucosal layer, with no evidence of intravascular cancer thrombus. The TNM stage was determined to be pT2N0M0. Immunohistochemical analysis revealed positivity for MUC1, MUC2, MUC5AC, MUC6, CK7, and CK19, and negativity for CK20 and CDX2, thereby supporting the diagnosis (**Figures 3B–D**). Additionally, postoperative next-generation sequencing (NGS) identified mutations in KRAS (Q61R in exon 3), TP53 (P58Qf*65 in exon 4), ROS1 (R77Q in exon 4 and T2052N in exon 39), and SDHA (V253G in exon 6). The patient had no pathogenic BRCA1/2 mutations and was microsatellite stable (MSS). Laboratory tests showed the following results: CA 19-9 19.15 U/ml (decreased from exceeding 1000 U/ml) (**Figure 2**) and CA 125 59.09 U/ml (increased from 10.98 U/ml). AFP and CEA were still at normal level on 2022/03/28.

The patient then received 8 cycles of GN chemotherapy over six months: Gemcitabine 1600 mg/m2 on days 1 and 8, Albumin-bound Paclitaxel 200 mg/m2 on days 1 and 8, C1D1, on 2022/04/15. Because of grade III neutropenia, the dose was adjusted to: Gemcitabine 1400 mg/m2 on day 1 and 8, Albumin-bound Paclitaxel 200 mg/m2 on day 1 and 8, C1D8-C8D8, from 2022/04/25 to 2022/09/23. The chemotherapy process went smoothly.

Note: conventional GN (modified AG): Albumin-bound paclitaxel 125mg/m2 IV drip Day 1 and 8 + Gemcitabine 1000mg/m2 IV drip Day 1 and 8, repeat every 3 weeks for 1 cycle.

**Figure 4:**
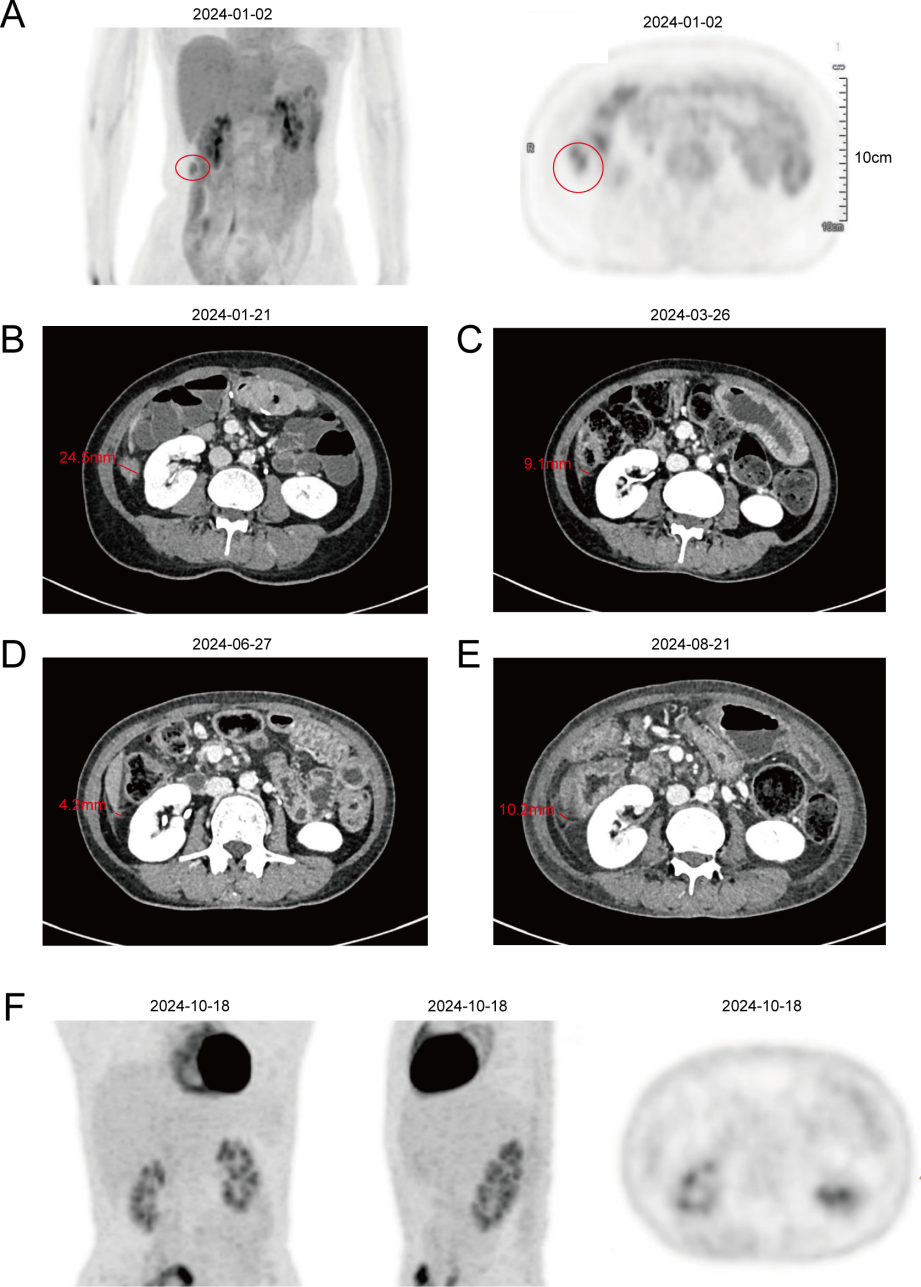
Imaging images of the patient after peritoneal metastasis. **(A)** PET/MR images of the patient. **(B–E)** CT images of the patient. **(F)** PET/CT images of the patient.

Serial imaging evaluations on March 08, June 07, August 30, and November 22, 2023, revealed no evidence of tumor recurrence. However, serum CEA levels began to rise on June 06, 2023 (**Figure 2**), and CA 19–9 levels subsequently increased to 36.3 U/ml on November 20, 2023, and 91.6 U/ml on January 10, 2024 (**Figure 2**). On January 02, 2024, PET/MR imaging demonstrated increased FDG uptake in the ascending paracolic gutter, consistent with peritoneal metastases (**Figure 4A**). A CT scan performed on January 21, 2024, revealed a patchy lesion with blurred margins at the lower edge of the right hepatic lobe and along the peritoneum adjacent to the ascending colon, measuring approximately 24.5 × 15.1 × 23.5 mm with moderate enhancement (**Figure 4B**).

The patient was enrolled in the HR070803–301 clinical trial (NALIRIFOX) and assigned to the experimental group. The regimen consisted of oxaliplatin 85 mg/m², HR070803 (liposomal irinotecan) 60 mg/m², L-5-methyltetrahydrofolate calcium 400 mg/m², and 5-FU 2400 mg/m² administered on days 1 and 15, repeated every 4 weeks for a total of 6 cycles (from January 16, 2024, to August 08, 2024). The chemotherapy was well tolerated.

After cycles 4 and 7, imaging evaluations indicated partial remission (PR); CT scans demonstrated a patchy lesion with blurred margins at the lower edge of the right hepatic lobe and the peritoneum adjacent to the ascending colon, with a maximum diameter of approximately 9.1 mm on March 26, 2024, and 4.2 mm on June 27, 2024 (**Figures 4C, D**). However, following cycle 6, imaging showed that the peritoneal metastatic lesions had increased in size, with a maximum diameter of about 10.2 mm on August 21, 2024 (**Figure 4E**). The patient was subsequently discharged from the clinical trial. After a 2-month interval without antitumor treatment, a PET/CT evaluation on October 18, 2024, revealed that the peritoneal metastatic lesions had disappeared, and the CA 19–9 level had decreased to 9.2 U/ml (**Figure 2**, **Figure 4F**). To date, no peritoneal metastatic lesions are detectable, and the CA 19–9 level remains within normal limits, indicating that the patient has achieved complete remission (CR).

## Discussion

Pancreatic ductal adenocarcinoma (PDAC) is one of the most aggressive malignancies, characterized by a dismal prognosis, rapid progression, and a high recurrence rate (12). Although the current standard of care—surgical resection combined with adjuvant chemotherapy—remains the mainstay of treatment, long-term survival continues to be limited by early metastatic spread and inherent resistance to conventional therapies (13). In the present case, the patient underwent a robotic-assisted laparoscopic pancreaticoduodenectomy shortly after diagnosis, followed by adjuvant chemotherapy using the GN regimen. This approach resulted in a remarkable 17-month tumor-free interval, highlighting the potential efficacy of our established treatment protocol for resectable PDAC.

A characteristic feature of PDAC is its propensity for rapid local infiltration, often involving tissues adjacent to the duodenum, liver, stomach, spleen, and major blood vessels. This infiltrative behavior complicates imaging interpretation, making it difficult to discern whether a lesion represents primary pancreatic cancer invading surrounding structures or an extrapancreatic malignancy extending into the pancreas (14). In our case, initial CT showed wall thickening of the duodenal bulb with indistinct margins and subtle enhancement changes; EUS revealed a duodenal mucosal mass with biopsy showing adenocarcinoma. However, based on imaging it was difficult to completely exclude pancreatic head carcinoma with duodenal invasion. These observations highlight the limitations of current imaging modalities in accurately diagnosing PDAC, particularly when the tumor invades neighboring tissues. According to the NCCN resectability criteria, important arteries and veins of this patient were not involved; therefore, even if the diagnosis were pancreatic head cancer, immediate surgery was appropriate. Consequently, enhancing early diagnostic capabilities—through advanced imaging techniques and the incorporation of molecular profiling—is essential to ensure patients receive timely and optimal treatment.

PDAC continues to exhibit a high recurrence rate despite aggressive multimodal treatments—including surgery, chemotherapy, and radiation therapy (2). Recurrence is often insidious, with patients remaining asymptomatic until the disease is advanced (14). Among the patterns of recurrence, peritoneal metastases pose a significant therapeutic challenge due to the inherent difficulties in delivering effective treatment to the peritoneal cavity, and they generally portend a poorer prognosis. In our case, after completing eight cycles of the AG regimen, a gradual increase in CA19–9 was noted—preceding the radiological detection of peritoneal metastases by approximately six weeks. Previous studies have demonstrated that CA19–9 elevations can be observed 2–3 months before recurrence is evident on imaging (15, 16). While current NCCN guidelines advocate for regular postoperative monitoring using both CA19–9 levels and imaging modalities (e.g., CT or MRI) (17), an elevated CA19–9 should serve as an early warning rather than a standalone indication for antitumor therapy. This observation raises several important considerations. First, the discordance between biochemical and radiological findings underscores the need for an integrated surveillance strategy that combines both approaches. Second, the management of peritoneal metastases may require novel or adjunctive therapeutic interventions beyond conventional chemotherapy. Future studies should explore the potential of advanced imaging techniques and emerging biomarkers, as well as investigate targeted therapies in the setting of early biochemical recurrence, to ultimately improve outcomes in PDAC.

Current guidelines recommend testing for BRCA1/2 and MSI H early in metastatic PDAC: BRCA mutated patients benefit markedly from platinum- based chemotherapy and PARP inhibitors, whereas MSI H accounts for ~1% of PDAC and may respond to immunotherapy. The patient had no pathogenic BRCA1/2 mutations and was microsatellite stable (MSS). Therefore, she lacked indications for PARP inhibitors or immune checkpoint blockade. Following enrollment in the HR070803–301 clinical trial, imaging after seven cycles of treatment revealed partial remission, underscoring the efficacy of the guideline-recommended first-line NALIRIFOX regimen for recurrent pancreatic cancer. Notably, this patient experienced a relapse after the post-surgery AG regimen but subsequently achieved CR with NALIRIFOX. Both the AG and NALIRIFOX regimens are currently utilized as first-line treatments for metastatic pancreatic cancer, and previous studies have indicated that NALIRIFOX may offer advantages in terms of progression-free survival (PFS) and overall survival (OS) (18). It is important to note that no intravenous agents specifically target peritoneal metastases. In this context, EMSO guidelines advocate for an individualized treatment approach, recommending either the AG or NALIRIFOX regimen based on the patient’s overall condition. Moreover, recent years have seen a surge in studies exploring therapeutic strategies for peritoneal metastases, including localized chemotherapy (e.g., intraperitoneal chemotherapy), targeted therapy, and immunotherapy, all of which have shown potential efficacy in select patients (10, 19, 20). These findings highlight the critical need to explore personalized and diversified therapeutic options in patients with recurrent PDAC, especially those with peritoneal metastases. Future research should aim to integrate novel treatment modalities with established regimens to optimize outcomes and further improve survival in this challenging patient population.

NALIRIFOX is a potent regimen and requires proactive management: First, antiemetic prophylaxis: Triple antiemetic prophylaxis using an NK1 receptor antagonist, a 5−HT3 antagonist and dexamethasone. Second, Hydration and electrolyte management: Intravenous fluids and close electrolyte monitoring reduce renal and neurotoxic effects. Third, Myeloid support: Administer prophylactic G−CSF and adjust dosage or interval based on haematologic toxicity. Forth, Patient education: Instruct patients on self−management of diarrhea and cold−induced peripheral neuropathy. The NAPOLI−3 trial showed that NALIRIFOX significantly prolonged overall survival and progression−free survival versus nab−paclitaxel plus gemcitabine in previously untreated metastatic PDAC (median OS 11.1 months vs 9.2 months; hazard ratio 0.83) (11). Toxicities mainly included gastrointestinal adverse events and hypokalaemia, whereas myelosuppression was relatively mild. With the above supportive measures, long−term treatment is feasible.

The most intriguing aspect of this case is that the patient’s peritoneal metastases appeared to progress on imaging after completion of the clinical trial treatment, yet CR was ultimately achieved without further antitumor therapy. This phenomenon—termed pseudoprogression—refers to the transient increase in lesion size on imaging due to tumor necrosis and inflammatory edema following effective antitumor treatment, rather than true disease progression (21, 22). In our patient, a rapid elevation of the CEA index was observed from the initiation of the NALIRIFOX regimen until after the final dose. Previous studies have reported that CEA levels may transiently rise during the initial phase of irinotecan treatment before declining significantly (23). These fluctuations, therefore, do not necessarily indicate true tumor progression but rather suggest effective treatment with resultant tumor necrosis and subsequent release of tumor markers into circulation (24). While pseudoprogression is well-characterized in immunogenic malignancies treated with immunotherapy, it remains exceedingly rare in pancreatic cancer patients receiving conventional chemotherapy. It is plausible that the NALIRIFOX regimen may induce a heightened inflammatory response or dynamic alterations in the TME (11, 25). Conventional size-based assessments may fail to differentiate between “false” enlargement due to inflammatory changes, edema, or necrosis and genuine tumor growth. To distinguish pseudoprogression from true progression, we need to combine special imaging and specific laboratory indicators. Functional imaging provides critical insights: perfusion MRI/CT often shows increased perfusion in pseudoprogression versus decreased perfusion in true progression, while DWI demonstrates reduced diffusion restriction in pseudoprogression compared to increased restriction in progressive disease. PET-CT further aids differentiation, with pseudoprogression typically exhibiting stable or decreased metabolic activity (26). Although CA19–9 may transiently elevate during pseudoprogression, sustained elevation >2× baseline strongly suggests true progression (15). Most notably, ctDNA dynamics demonstrate high clinical utility, with elevated levels correlating strongly with true progression (27). Integration of functional imaging with serial biomarker assessment improves diagnostic accuracy and should be considered in ambiguous cases.

The selection of imaging modalities for monitoring recurrence and metastasis in pancreatic cancer is particularly critical. We compared imaging modalities for recurrence surveillance: CT: Rapid and wide−field scanning makes CT the first−line tool for postoperative follow−up, especially to detect lung metastases and assess anatomical structures. MRI: High soft−tissue contrast and incorporation of DWI or dynamic sequences allow better differentiation between local recurrence and scar tissue; MRI is particularly sensitive for liver and pancreatic bed lesions. PET/CT: Provides metabolic information to detect small metastases not evident on conventional imaging and is useful when tumour markers rise but CT/MRI are normal. PET/MR: Combines metabolic PET data with the anatomical and functional advantages of MRI, yielding more accurate assessment in complex regions (e.g., postoperative pancreatic bed or liver) (28).

Different tumour markers undergo different clearance and metabolic pathways, so their decline rates may differ. CA19–9 commonly rises 2–6 months before relapse and falls rapidly after complete response. CEA may decline more slowly owing to hepatic metabolism. In our case, CEA remained mildly above normal but showed a downward trend; the patient also had mildly elevated liver enzymes, which might have delayed CEA clearance.

Given the high recurrence risk of PDAC, structured surveillance is essential. The current NCCN guidelines (expert consensus) recommend reviewing history, physical examination, CA19−9 and contrast−enhanced CT every 3–6 months during the first two years after resection, then annually thereafter. Because our patient experienced recurrence, we propose a more intensive schedule: Follow−up frequency: CA19−9, CEA, liver and kidney function tests and thoraco−abdominal contrast−enhanced CT every 3–6 months. Escalation of imaging: When tumor markers rise or CT shows suspicious lesions, proceed to PET−CT or PET−MRI to detect occult recurrence. Duration: Although most recurrences occur within two years, late recurrences have been reported. We therefore plan at least five years of surveillance and will extend follow−up depending on the patient’s clinical status.

The three cases reviewed focus on patients with BRCA2 mutation/high MSI and those who have received treatments such as mFOLFIRINOX, GN, pembrolizumab, and surgical resection post-metastasis (29–31). In contrast to these cases, our case highlights a pancreatic cancer patient with no specific mutation type. Furthermore, this case illustrates the entire diagnostic, surgical, adjuvant therapy, and postoperative monitoring pathway for pancreatic cancer. Key aspects include: 1) the emphasis on the superiority of NALIRIFOX in first-line treatment for metastatic pancreatic cancer, 2) a discussion of the rare occurrence of pseudoprogression in pancreatic cancer, and 3) a comprehensive evaluation of the diagnostic and therapeutic approach in the management of pancreatic cancer. This case provides important clinical insights, particularly on the necessity of integrating multiple imaging and laboratory indicators for the accurate differentiation of pseudoprogression from true disease progression.

## Data Availability

The raw data supporting the conclusions of this article will be made available by the authors, without undue reservation.

## Supplementary material

Supplementary Figure 1 Preoperative EUS images of the patient. **(A, B)** EUS images of the patient. **(C–F)** Endoscopic images of the patient.
