# Supplementary figures and images for: Unexpected complete remission after recurrence in pancreatic cancer: a case report on comprehensive multimodal therapy
Source: Front Oncol. 2025 Oct 17;15:1582673. doi: 10.3389/fonc.2025.1582673 (PMC12575098; doi:10.3389/fonc.2025.1582673)

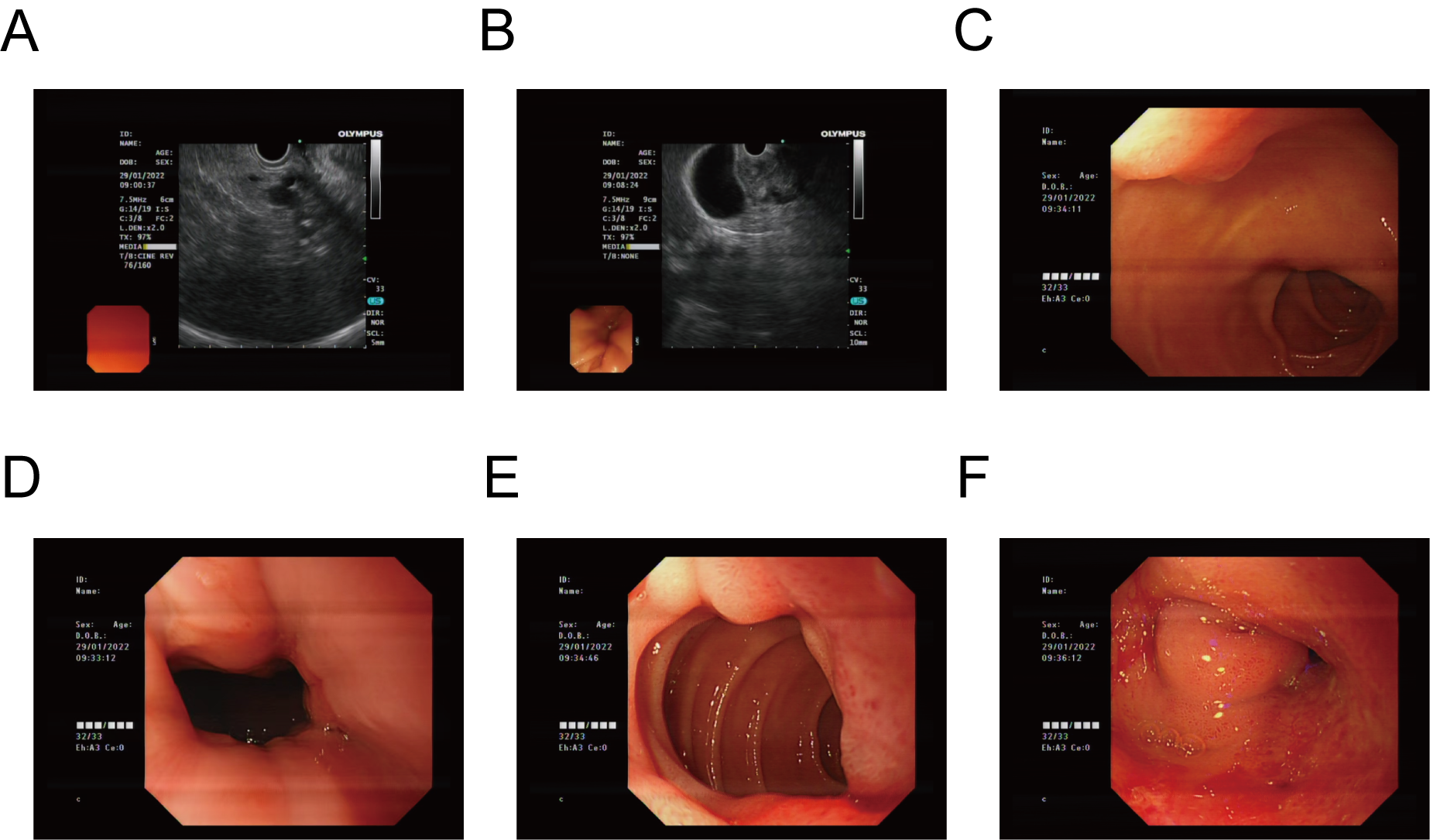

Supplement: Supplementary Figure 1 — Preoperative EUS images of the patient. (A, B) EUS images of the patient. (C–F) Endoscopic images of the patient. [file Image1.tif]
